# Promotion of Mental Health Rehabilitation in China: Community-Based Mental-Health Services

**DOI:** 10.17650/2712-7672-2020-1-1-21-27

**Published:** 2020-12-04

**Authors:** Youwei Zhu, Xu Li, Min Zhao

**Affiliations:** Shanghai Mental Health Centre, Shanghai Jiao Tong University School of Medicine; Shanghai Key Laboratory of Psychotic Disorders

**Keywords:** China, community-based mental health service, recommendations, Китай, амбулаторная психиатрическая служба, рекомендации

## Abstract

Community-based mental health services are important for the treatment and recovery of patients with mental health disorders. The Chinese government has made the establishment of a highly eﬃcient community-based health service an enduring priority. Since the 1960s, community-based mental health services have been developed in many Chinese cities and provinces. National policies, including mental health regulations and ﬁve-year national mental health working plans, have been issued to support the development of quality of mental health services. The accessibility and eﬃciency of community-based mental health services are now highly promoted to community residents.

According to the National Standards for Primary Public Health Services, community-based mental health services are one of the most important components of primary public health services. They are mainly provided via Community Health Service Centres (CHCs), by a combination of general practitioners, public health physicians, nurses and social workers. Patients receive individualized and continuous health services according to their rehabilitation status. These services include regular physical examination, health education, rehabilitation guidance, social function rehabilitation training, vocational training and referral services; family members also receive care and psychological support. Future work will focus on expanding mental health service coverage and usage, increasing awareness of mental health and decreasing stigma, and strengthening service capability to establish an integrated model to enhance the overall eﬃciency of mental health services.

## THE MENTAL HEALTH SERVICE SYSTEM IN CHINA

Mental disorders are one of the most serious public health challenges, affecting individuals, their families and society. A recent national mental health survey in China showed that the lifetime prevalence of mental disorders was 16.6%, with anxiety disorders, mood disorders, substance use disorders, impulse control disorders and schizophrenia among the most common [1]. Nearly 20% of the burden of diseases in China is caused by mental disorders and suicide [2]. This growing burden highlights the urgent need for an effective mental health service system. Nowadays, nearly 90% patients are living and receiving medical treatments in their communities, which has implications for the development of high-quality mental health services, particularly those based in the community.

The Chinese government has long been committed to providing better quality and more integrated mental health services, aiming at improving the efficiency and continuity of both hospital-based and community-based mental health services. Mental health laws, related regulations and national mental health working plans have also been enacted in recent decades in order to facilitate the development of mental health services. Since the 1960, there has been an integrated three-tier system that includes inpatient, outpatient and community services [3]. In this article, we will mainly focus on community-based mental health services.

### Mental health-related policy In China

The first National Mental Health Plan (2002-2010) was signed by the Ministries of Health, Public Security and Civil Affairs, and the China Disabled Persons' Federation (CDPF) in 2002. It aimed to establish an effective system of mental health care, led by the government, with the participation and cooperation of other sectors [4]. The service model was led by psychiatric hospitals, supported by departments of psychiatry in general hospitals, community-based health facilities and rehabilitation centres.

On 26th October 2012, The Mental Health Law of the People's Republic of China was enacted to develop the field of mental health, standardize mental health services and guarantee the legal rights and interests of people with mental disorders [5]. This Law mandated that urban community health centres, rural township health centres and rural village health clinics should establish a registry of people with severe mental disorders, periodically follow up such people who live at home, instruct patients about the use of medication and rehabilitation, and educate guardians about mental health and care of the mentally ill.

Following this Law, the National Mental Health Plan (2015-2020) was proposed by the State Council. It contained more specific aims and requirements to improve the mental health system and to develop mental health services [6].

### 1.2 A three-tier mental health system

An integrated, three-tier hospital-community service model has been established and has become a crucial component in the reform of China's mental health services [7]. Mental health services are mainly provided by psychiatrists, psychiatric nurses, social workers and clinical psychologists in mental health centres, by general practitioners and community nurses in community health centres and by social workers, clinical psychologists, rehabilitation therapists and occupational therapists in other government or social organizations. These services cover inpatient and outpatient treatment, hospital and community rehabilitation, health education, psychotherapy and vocational rehabilitation. Patients with mental illnesses can seek mental health services both in mental health centres and community health centres. The government has clearly defined the responsibilities of different medical institutions at all levels in the model. Psychiatric hospitals are mainly in charge of medical treatment for severe mental disorders, through inpatient and outpatient treatment, hospital-based rehabilitation and health education. When a patient's condition has stabilized, they are referred to CHCs, which are responsible for rehabilitation and health education. If the patient is willing to accept follow-up services, mental health service providers will provide patients and their guardians advice on how to maintain stability. If the patient is relatively stable, they may attend community-based rehabilitation facilities. These facilities provide services including antipsychotic maintenance therapy, behaviour therapy, social skills training, vocational rehabilitation and family education. If the patient relapses, they are referred to hospital.

### The mental health workforce and resources

The mental health workforce is a key component of the quality and efficiency of mental health services. In the past, mental health services were mainly hospital-based, delivered by psychiatrists or psychiatric nurses in psychiatric hospitals. The capacity of community-based services was limited, as there were far fewer mental health professionals working in community health centres, which also severely affected the continuity of mental health services. To meet this challenge, governments at all levels were committed to improving community-based mental health services and strengthening professional capacity and human resources.

In 2002, there were only 13,397 registered psychiatrists [8], but by the end of 2016, this number had risen to 31,910 [9] (2.31/100,000 population; above the average of 2.11/100,000 in upper-middle income countries) [10]. By the end of 2015, there were about 1.2 million certified psychological counsellors, however, only 0.03-0.04 million were involved in psychological counselling work, part-time or full-time and there were only about 5000 psychotherapists [11]. The number of mental health social workers is not clear. By the end of 2016, there were 1,650 psychiatric hospitals, containing 297,637 beds (21.5/100,000 population; below the average of 24.3/100,000 population in upper-middle income countries). The average length of inpatient stay is 51.7 days [9]. The quality and coverage of mental health services have been greatly improved.

## COMMUNITY-BASED MENTAL HEALTH SERVICES: FOUNDATIONS AND DEVELOPMENT

Following the first National Mental Health Meeting in 1958, community-based mental health rehabilitation work started in Beijing, Shanghai, Hunan, Sichuan, Jiangsu and Shandong provinces, before gradually expanding to other places in China. As the vast majority of patients receive treatments and therapies in the communities where they live, the provision of comprehensive, continuous and coordinated mental health care services is very important for patient recovery. The Chinese government has promised to make continuous efforts to develop a better community-based hospital-community integration service model in order to meet individual need for qualified mental health services in communities.

### Service development 2002-2018

In 2002, the State Council issued the National Mental Health Working Plan (2002-2010), which emphasized the building of a better mental health service delivery system for the prevention, treatment and rehabilitation of mental disorders, based on medical institutions, communities and families. In 2012, the Mental Health Law mandated that CHCs must provide technical support to help residents' committees to provide mental health education to all residents living in the community. They are also responsible for keeping records of patients with severe mental disorders, follow-up services, medication guidance, rehabilitation training and mental health and nursing knowledge education for guardians [12]. In 2014, the updated National Mental Health Working Plan (2015-2020) was implemented. This proposed a clear aim: continuing to improve the mental health services for prevention, treatment and rehabilitation of mental disorders to meet individual need for mental health services.

In 2018, 10 related departments jointly announced the Pilot Working Plan for the Construction of a National Psychosocial Service System. This policy highlighted the importance of improving the continuity of community-based mental health services. It encouraged treatment and rehabilitation information-sharing and the use of information technology [13]. Also in 2018, the Ministry of Civil Affairs, the Ministry of Finance, Health and Family Planning Commission and the CDPF jointly issued Guidance on Accelerating the Development of Community Rehabilitation Services for Mental Disorders. These two documents mean that patients can receive health services covering disease prevention, treatment, recovery and relapse prevention in their communities [14]. Patients can begin their rehabilitation treatment as soon as possible with continuity between hospital and community rehabilitation.

### The structure and function of community-based mental health services

In 2004, the Central Government Support for the Local Management and Treatment of Severe Mental Illnesses Project ('686 Programme') [15] was implemented to expedite the process of exploring and establishing a better hospital-community integration service model. In order to further standardize service quality and scope, in 2009 the National Standards for Primary Public Health Services (First Edition) were issued. As mental health is one of important public health issues, mental health services, especially for severe mental illnesses, were included [16].

These two policies aimed to promote and standardize community-based mental health services via CHCs in urban and rural areas. Governments at all levels have led the construction and development of the community-based mental health service system, with CHCs, psychiatric hospitals and Centres for Disease Control and Prevention (CDCs) for mental health. CHCs are obliged to deliver basic public health services, i.e., community-based health services are mainly provided by general practitioners, public health physicians and nurses working in these centres. To alleviate the shortage of human resources, psychiatrists and nurses from mental health centres are responsible for providing technical support to this workforce. CDCs are largely responsible for community mental health service quality control and supervision, the introduction of new interventions and rehabilitation techniques for mental health services, information management and disease surveillance for patients with severe mental disorders.

### Community Health Service Centres

In accordance with the requirements of the 686 Programme and the National Standards for Primary Public Health Services, community-based mental health services have many responsibilities, including: establishing individual health records, health assessment, annual physical examination, follow-up after hospitalization or outpatient treatment at least four times a year, appropriate intervention and prevention measures according to severity of illness, referral services, health education for patients and their guardians, rehabilitation guidance and psychological support for family members. Importantly, these services are voluntary, and all expenditure is covered by government [17].

After outpatient or inpatient treatment in psychiatric hospitals, patients are referred to their CHC, where they (or their guardian) are asked to consent to follow-up services. Health assessment, diagnosis and treatment information are added to their health record. Patients can receive a follow-up service at least four times a year according to their rehabilitation progress. These include assessments of relapse risk, mental state, physical illnesses, social function, medication and laboratory tests, which inform individuallzed health services tailored to patients' needs. Regular physical examination, health education, rehabilitation guidance, social function rehabilitation training, vocational training and referral services; plus care and psychological support for family members are also included. To facilitate convenience and efficiency, some of the services can be delivered at home or at the CHCs by experienced general practitioners and community psychiatric nurses. If a patient's condition is found to be unstable, referral to mental health centres for hospitalization or outpatient treatment, or hospital-based home care, are provided.

Rehabilitation is one of crucial goals for community-based mental health services. Patients with stable conditions can choose to receive a variety of services to promote rehabilitation. Many provinces and cities have established community rehabilitation institutes, supported by many organizations including CDPF, the Civil Affairs Department, CHCs and social work organizations. Rehabilitation treatments gradually shift focus from disease to people; from symptom elimination to recovery of social functions. Individualized services are provided for patients at different stages, to support rehabilitation in cognitive ability, life skills, hobbies, social ability, vocational skills and to improve overall quality of life.

## OUTCOMES AND IMPACTS OF COMMUNITY MENTAL HEALTH SERVICES

Historically, mental health services were mainly hospital-based, but reform and development of community-based health services has seen the establishment of an integrated hospital-community model.

Decades of development have led to substantial improvements in the quality of medical treatment and rehabilitation therapies, the continuity of mental health services, the coverage of patients with severe mental health disorders, the immediate treatment rate of patients, medicine-taking rate, treatment adherence and support for family members. There are now more than 35,000 CHCs and more than 1,600 institutions that provide high-quality mental health rehabilitation services. By the end of the 2016, there were more than 5 million patients with severe mental health disorders registered with community-based mental health services. More than 90% of these patients had opted to receive support and 75% received regular follow-up services [18]. Other important indicators, such as (regular) medication-taking rate and number of patients in a stable condition, are also rising every year [19]. The construction of an electronic health records system facilitates the transfer of information about patients' medication, treatment and rehabilitation therapies to general practitioners after hospital discharge, meaning that patients receive a more individualized treatment and rehabilitation service. Community residents, especially those with severe mental health disorders, benefit from much-improved access to mental health services. The median radius of the mental health service network of each service site exceeds 184 km, which covers more than 90% of patients with severe mental disorders [18].

## CHALLENGES AND FUTURE DIRECTIONS

The Chinese government has made the establishment of a highly efficient community-based health service an enduring priority. However, there are still many challenges that need to be addressed.

### Mental health service coverage and usage

China is a huge country; unequal distribution of mental health service resources hinders the capacity of community-based mental health services. Despite the rapid development of mental health services in recent years, resources are still concentrated in rich coastal or eastern areas and most are allocated to patients with severe mental health disorders. Studies have indicated that utilization rates of community-based mental health services are not high enough [20], as individuals still prefer mental health centres or general hospitals when seeking medical treatment. Efforts to expand coverage and accessibility of mental health services in communities is encouraged by health reform.

### Mental health awareness and stigma

As in other countries, lack of knowledge and stigma surrounding mental health disorders mean that people are reluctant to seek mental health service resources [21]. Health education to improve knowledge of mental disorders seems insufficient to address this problem [22]. Rather, there is a need for more anti-stigma interventions and systematic health education focusing on changing attitudes, including those of mentally ill patients, to help reduce stigma and improve mental health literacy [23].

### Mental health service capability

The number of medical practitioners in CHCs is a key element in determining the quantity, quality and effectiveness of mental health service delivery and capability. Currently, there are not enough psychiatrists and other mental health professionals in CHCs to meet the needs of all residents in communities. Training of psychiatrists and nurses has already accelerated in many places. Psychiatrists in tertiary hospitals should be encouraged to obtain a multi-site licence to practise in CHCs, so that they can enhance service capability and provide technical guidance. Teamwork among practitioners from psychiatric hospitals, CHCs and social work organizations is also recommended to maximize existing service capabilities.

In summary, establishing an integrated service model is crucial for the development of community-based mental health services. The hospital-community integration service model has become the most common and recommended model. It has been developed significantly and plays an important role in the mental health service system. However, closer coordination among mental health centres, CHCs and related government sectors is still needed. The coordination mechanisms between related services providers also need to be further strengthened to provide better community mental health services to meet the mental health needs of all individuals in China.

## Authors contribution

Zhu Youwei and Li Xu: literature researching and article writing. Zhao Min: data checking, literature evaluation and language editing.

## Conflict of interest

The authors declare that there are no known conflicts of interest associated with this publication.

## Funding

This study was funded by support from the National Key R&D Programme of China (2017YFC1310400), National Nature Science Foundation of China (Ul 502228, 81501148), Shanghai Municipal Health and Family Planning Commission (2014ZYJB0002), Shanghai Health and Family Planning Commission Clinical Research Project (20184Y0134, 20184Y0152), Programme of Shanghai Academic Research Leader (17XD1403300), Shanghai Key Laboratory of Psychotic Disorders (13DZ2260500), Qihang Project of Shanghai Mental Health Centre (2018-QH-02) and Shanghai Mental Health Centre (2017-YJ-12). The funders have no role in study design, data collection and analysis, decision to publish, or preparation of the manuscript.

## Ethics approval and consent to participate

All procedures followed were in accordance with the ethical standards of the Norwegian National Committee for Research Ethics in the Social Sciences and the Humanities and with the Helsinki Declaration of 1975, as revised in 2000.

## Abbreviations

CDPF: China Disabled Persons' Federation

CHCs: Community Health Service Centres

CDCs: Centres for Disease Control and Prevention
